# Genome Characterization of the Oleaginous Fungus *Mortierella alpina*

**DOI:** 10.1371/journal.pone.0028319

**Published:** 2011-12-08

**Authors:** Lei Wang, Wei Chen, Yun Feng, Yan Ren, Zhennan Gu, Haiqin Chen, Hongchao Wang, Michael J. Thomas, Baixi Zhang, Isabelle M. Berquin, Yang Li, Jiansheng Wu, Huanxin Zhang, Yuanda Song, Xiang Liu, James S. Norris, Suriguga Wang, Peng Du, Junguo Shen, Na Wang, Yanlin Yang, Wei Wang, Lu Feng, Colin Ratledge, Hao Zhang, Yong Q. Chen

**Affiliations:** 1 State Key Laboratory of Food Science and Technology, School of Food Science and Technology, Jiangnan University, Wuxi, People's Republic of China; 2 TEDA School of Biological Sciences and Biotechnology, Nankai University, Tianjin Economic-Technological Development Area, Tianjin, People's Republic of China; 3 Tianjin Research Center for Functional Genomics and Biochip, Tianjin Economic-Technological Development Area, Tianjin, People's Republic of China; 4 Key Laboratory of Molecular Microbiology and Technology of the Ministry of Education, TEDA School of Biological Sciences and Biotechnology, Nankai University, Tianjin, People's Republic of China; 5 Department of Cancer Biology, Wake Forest School of Medicine, Winston-Salem, North Carolina, United States of America; 6 Department of Biochemistry, Wake Forest School of Medicine, Winston-Salem, North Carolina, United States of America; 7 Department of Microbiology and Immunology, Medical University of South Carolina, Charleston, South Carolina, United States of America; 8 Tianjin Biochip Corporation, Tianjin Economic-Technological Development Area, Tianjin, China; 9 Department of Biological Sciences, University of Hull, Hull, United Kingdom; Tulane University Health Sciences Center, United States of America

## Abstract

*Mortierella alpina* is an oleaginous fungus which can produce lipids accounting for up to 50% of its dry weight in the form of triacylglycerols. It is used commercially for the production of arachidonic acid. Using a combination of high throughput sequencing and lipid profiling, we have assembled the *M. alpina* genome, mapped its lipogenesis pathway and determined its major lipid species. The 38.38 Mb *M. alpina* genome shows a high degree of gene duplications. Approximately 50% of its 12,796 gene models, and 60% of genes in the predicted lipogenesis pathway, belong to multigene families. Notably, *M. alpina* has 18 lipase genes, of which 11 contain the class 2 lipase domain and may share a similar function. *M. alpina*'s fatty acid synthase is a single polypeptide containing all of the catalytic domains required for fatty acid synthesis from acetyl-CoA and malonyl-CoA, whereas in many fungi this enzyme is comprised of two polypeptides. Major lipids were profiled to confirm the products predicted in the lipogenesis pathway. *M. alpina* produces a complex mixture of glycerolipids, glycerophospholipids and sphingolipids. In contrast, only two major sterol lipids, desmosterol and 24(28)-methylene-cholesterol, were detected. Phylogenetic analysis based on genes involved in lipid metabolism suggests that oleaginous fungi may have acquired their lipogenic capacity during evolution after the divergence of Ascomycota, Basidiomycota, Chytridiomycota and Mucoromycota. Our study provides the first draft genome and comprehensive lipid profile for *M. alpina*, and lays the foundation for possible genetic engineering of *M. alpina* to produce higher levels and diverse contents of dietary lipids.

## Introduction

**Figure 1:**
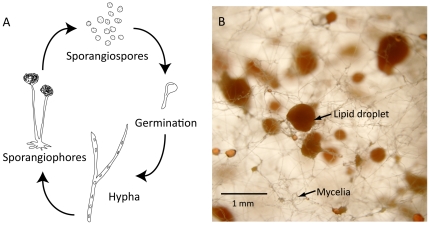
Asexual lifecycle and lipid staining of M. alpina. (**A**) Asexual lifecycle of the fungus. Haploid cells form sporangiophores, and sporangiospores germinate to hypha. (**B**) Fungal culture grown on PDA plate stained with 0.5% triphenoltetrazolium chloride. Lipid droplets are stained brown.

Single cell oils have garnered great interest in recent years as a source of both energy and dietary fat. *Mortierella alpina* belongs to the subphylum of Mucoromycotina [1]. In its haploid life cycle, hyphae germinate from sporangiospores and then form sporangiophores (**Figure 1A**). Lipids accumulate inside the hyphae and, over time, form lipid droplets on the mycelia (**Figure 1B**). *M. alpina* can produce lipids amounting up to 50% of its dry weight [2], [3], [4], mostly composed of triacylglycerol with high quantities of arachidonic acid (AA; 20:4n-6) [5], [6]. It is used for the industrial production of AA for incorporation into infant formulas with a record of complete safety [6], [7], [8], [9].

Unlike saturated fatty acids (SAFA) and mono-unsaturated fatty acids (MUFA), mammals cannot synthesize *de novo*, omega-6 or omega-3 polyunsaturated fatty acids (PUFA). Therefore, PUFA are essential fatty acids for human health and must be acquired via diet [10], [11], [12]. Fungi, however, such as *M. alpina*, can synthesize SAFA, MUFA, and PUFA *de novo*, and due to its high lipid content, provides an interesting model for studying lipid metabolism.

Some of the genes necessary for lipid synthesis in *M. alpina* have been cloned and partially characterized [13], [14], [15], [16], [17], and several biochemical reactions have been studied in detail [6], [18]. However, the molecular mechanism of efficient lipid biosynthesis is still not well understood in oleaginous fungi in general and in *M. alpina* in particular. The purpose of current study was to obtain *M. alpina* genome and transcript information, map its lipid metabolic pathway and analyze major lipid species, in order to better understand the mechanism of its efficient lipid biosynthesis.

## Results

### Features of the *M. alpina* genome

In the present study, we have assembled the *M. alpina* genome, annotated its transcripts, analyzed its mitochondrial genome, and determined its relationship to other oleaginous fungi. The genome of *M. alpina* ATCC#32222 was sequenced with 31.75-fold coverage (**Table S1**) and the sequence deposited in public genome databases (DDBJ/EMBL/GenBank accession ADAG00000000, first version ADAG01000000). The assembly contains a total contig length of 38.38 Mb with a GC content of 51.72% (**Table 1**). Comparison between the genome assembly and EST sequences shows that 99.94% of the EST sequences can be aligned to the assembly, covering 99.16% of the total length of EST sequences. This confirms that the current assembly represents more than 99% of the coding region of the genome.

**Table 1:** Features of the *M. alpina* genome.

**General features**	
**Size of assembled genome (Mb)**	**38.38**
Number of scaffolds larger than 2 kb	476
GC content (%)	51.72
Length of classified repeats (%)	1.76
**Number of predicted gene models**	**12796**
Average gene length (bp)	1847
Average transcript length (bp)	1504
Number of single-exon genes	3311
Average number of introns per multi-exon gene	3.32
Average exon size (bp)	435
Average intron size (bp)	140
Number of transposase-related genes	135
Number of tRNA genes	228
**Properties of predicted gene models**	**No. of genes**
NR alignment	8552
KEGG alignment	8382
KOG/COG assignment	7066
EC assignment	3290
KO assignment	6415
GO assignment	5800
InterPro signature	8740
Signal peptide	2041
Transmembrane domain	2286

**Figure 2:**
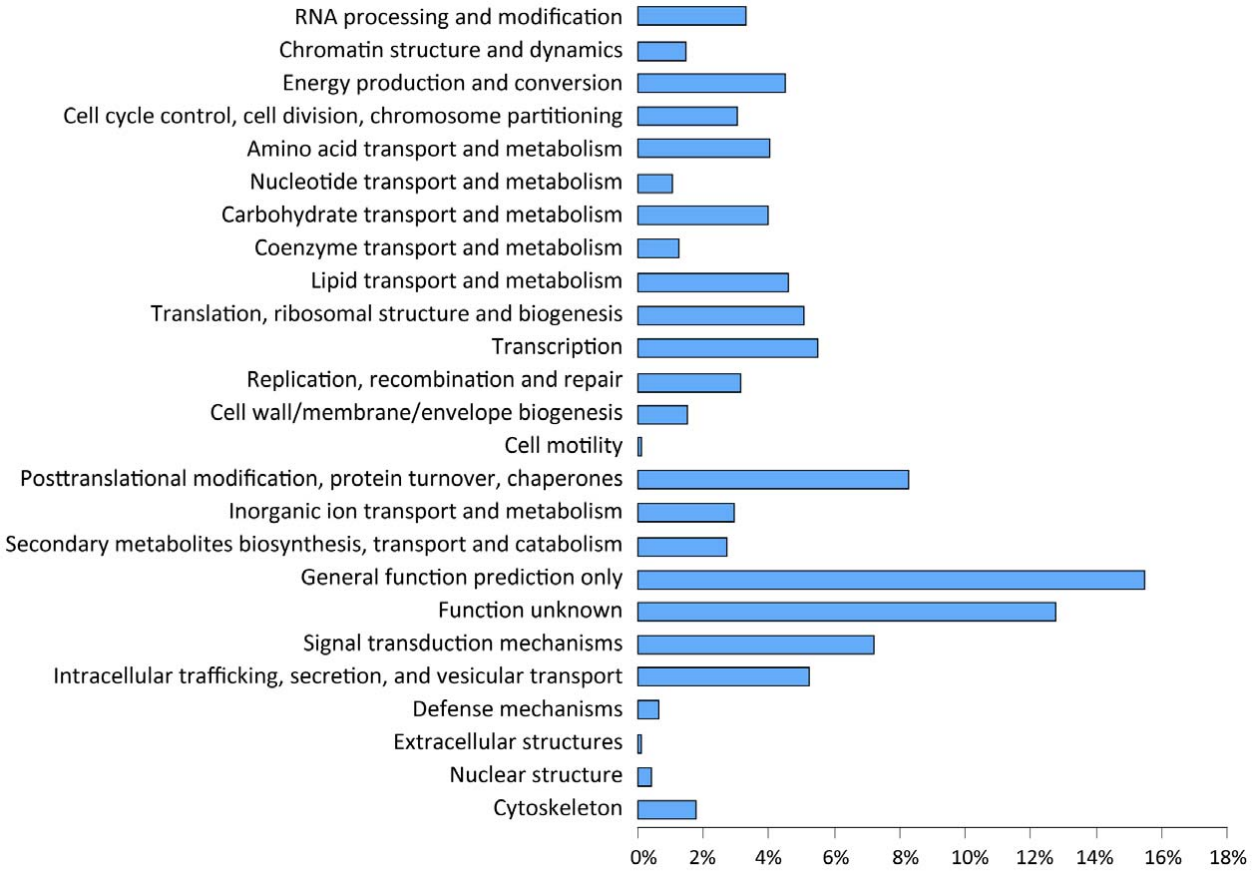
Distribution of predicted *M. alpina* proteins among functional groups. Gene prediction and annotation were performed as described in the Materials and Methods. KOG category assignment is shown. X-axis represents percent of predicted gene models.

Annotation of the assembled genome sequence generated 12,796 gene models with an average transcript length of 1.5 kb. There is an average of 3.32 introns per multi-exon gene and 26% of the predicted genes are single-exon transcripts. The average exon size is 435 bp and the average intron size is 140 bp. Approximately 33% of the predicted genes encode proteins with no homologs in the NR protein databases. Among those genes with homologs in other organisms, 8,382 genes were mapped to the KEGG database (**Table 1**). The 12,796 predicted genes can be classified into 25 functional categories with approximately 4% of the predicted genes belonging to the lipid transport and metabolism category (**Figure 2**). Although no novel lipogenic genes were identified, sequencing revealed that the genome of *M. alpina* has a high degree of gene duplications, with approximately 50% of gene models belonging to multigene families (**Table 2**). Fungal genome comparison indicates that species in the Mucoromycotina subphylum have 50–70% genes in multigene families, whereas species in the Ascomycota, Basidiomycota and Chytridiomycota have 20–60% genes in multigene families (**Table 2**).

**Table 2:** Comparative analysis of multi-gene families.

Species	Genes in the genome	Genes in multigene family (%)
**Mucoromycotina**		
*Mucor circinelloides* CBS277.49	11,719	72.00
*Rhizopus oryzae* 99–880	17,459	63.66
*Mortierella alpina* ATCC32222	12,796	50.34
**Ascomycota**		
*Nectria haematococca* MPVI	15,707	62.46
*Fusarium oxysporum* Fo5176	17,817	62.35
*Aspergillus niger* ATCC1015	10,950	55.25
*Trichoderma virens* Gv29-8	11,643	52.57
*Gibberella moniliformis* 7600	14,169	49.79
*Trichoderma atroviride* P1	11,100	49.18
*Aspergillus fumigatus* Af293	10,148	47.88
*Aspergillus clavatus* NRRL 1	9,121	43.57
*Aspergillus oryzae* RIB40	12,074	43.56
*Schizosaccharomyces japonicus* yFS275	10,046	43.36
*Trichoderma reesei* QM6a	9,116	42.63
*Schizosaccharomyces cryophilus* OY26	5,178	40.71
*Magnaporthe grisea* 70-15	14,010	40.19
*Aspergillus nidulans* FGSC A4	9,541	39.89
*Gibberella zeae* PH-1	11,656	38.98
*Neosartorya fischeri* NRRL 181	10,406	38.75
*Saccharomyces cerevisiae* S288c	5,880	36.87
*Aspergillus niger* CBS513.88	14,102	35.37
*Aspergillus fumigatus* A1163	9,630	35.28
*Vanderwaltozyma polyspora* DSM70294	5,336	34.50
*Scheffersomyces stipitis* CBS6054	5,816	31.31
*Schizosaccharomyces pombe* 972h-	5,003	30.68
*Candida glabrata* CBS138	5,191	30.65
*Botryotinia fuckeliana* T4	16,360	30.62
*Yarrowia lipolytica* CLIB122	6,472	30.05
*Debaryomyces hansenii* CBS767	6,324	29.90
*Podospora anserina* S mat+	10,272	29.51
*Candida albicans* SC5314	6,017	28.04
*Kluyveromyces lactis* NRRL Y-1140	5,335	23.41
*Sclerotinia sclerotiorum* 1980 UF-70	14,446	22.48
*Botryotinia fuckeliana* B05.10	16,389	21.58
*Neurospora crassa* OR74A	9,824	21.23
*Ashbya gossypii* ATCC10895	4,725	20.53
**Basidiomycota**		
*Laccaria bicolor* S238N-H82	18,215	53.82
*Phanerochaete chrysosporium* RP78	10,048	46.20
*Cryptococcus neoformans var. neoformans* B-3501A	6,273	27.02
*Ustilago maydis* 521	6,538	23.05
*Malassezia globosa* CBS7966	4,286	19.18
**Chytridiomycota**		
*Batrachochytrium dendrobatidis* JAM81	8,700	49.33

The number of transfer RNA (tRNA) units is estimated at 228 (**Table 1**). Repetitive elements that can be explicitly classified account for 1.76% of the genome sequence (**Table S2**), a relatively low level compared with other sequenced fungi, which range between 1 and 20%. A high level of repetitive elements may accelerate species evolution.

**Figure 3:**
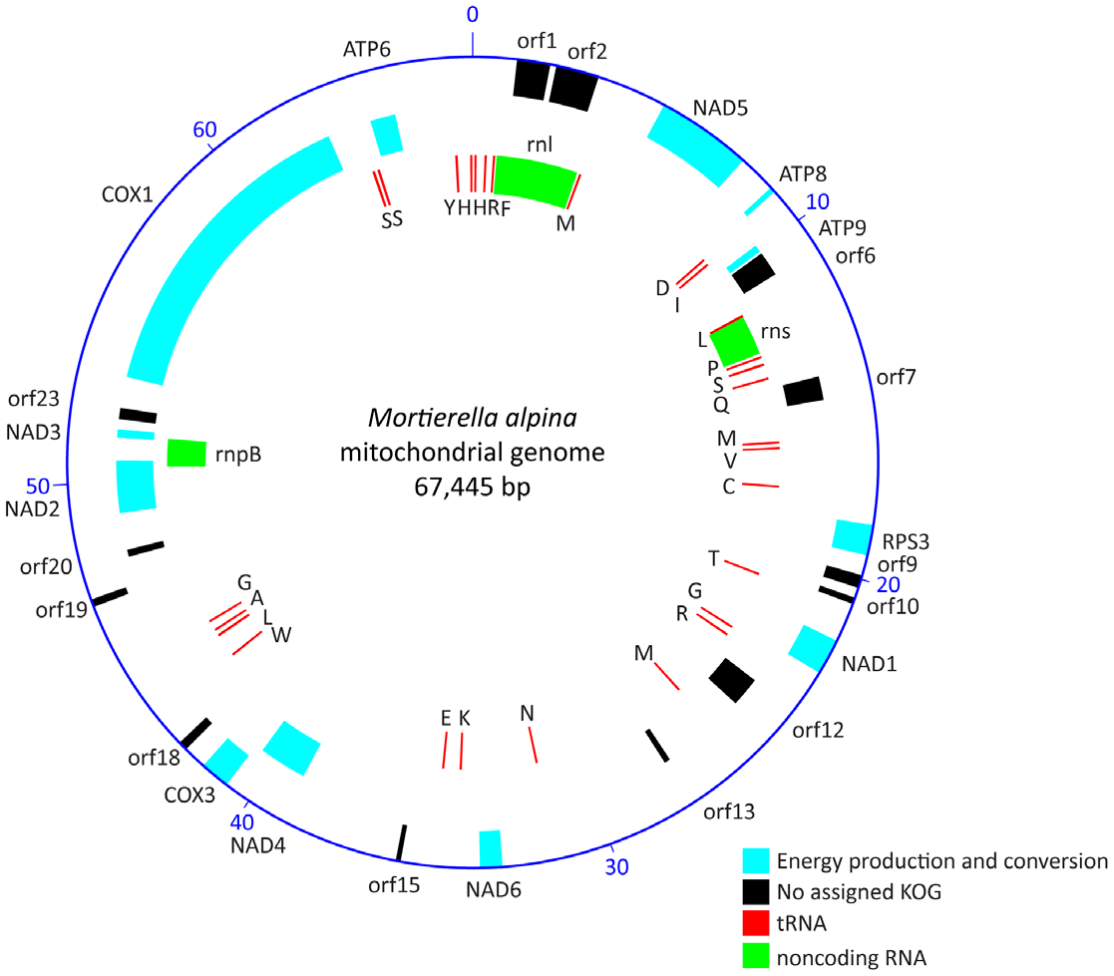
Map of *M. alpina* mitochondrial genome. The outer circle represents the scale in kb. From the outside in, circles 1 and 2 indicate the location of ORF transcribed in a clockwise and counter-clockwise direction, respectively. Transcripts were predicted according to KOG categories. Circle 3 depicts tRNA and noncoding RNA.

The mitochondrial genome of *M. alpina* measures 67,445 bp and presents 36.2% sequence homology with the mitochondrial genome of *M. verticillata* [19]. The *M. alpina* mitochondrial genome encodes 28 tRNAs, 3 noncoding RNAs (rnl, rns and rnpB), 12 known proteins (NAD1, NAD2, NAD3, NAD4, NAD5, NAD6, ATP6, ATP8, ATP9, RPS3, COX1, COX3) and 13 uncharacterized proteins (**Figure 3**).

**Figure 4:**
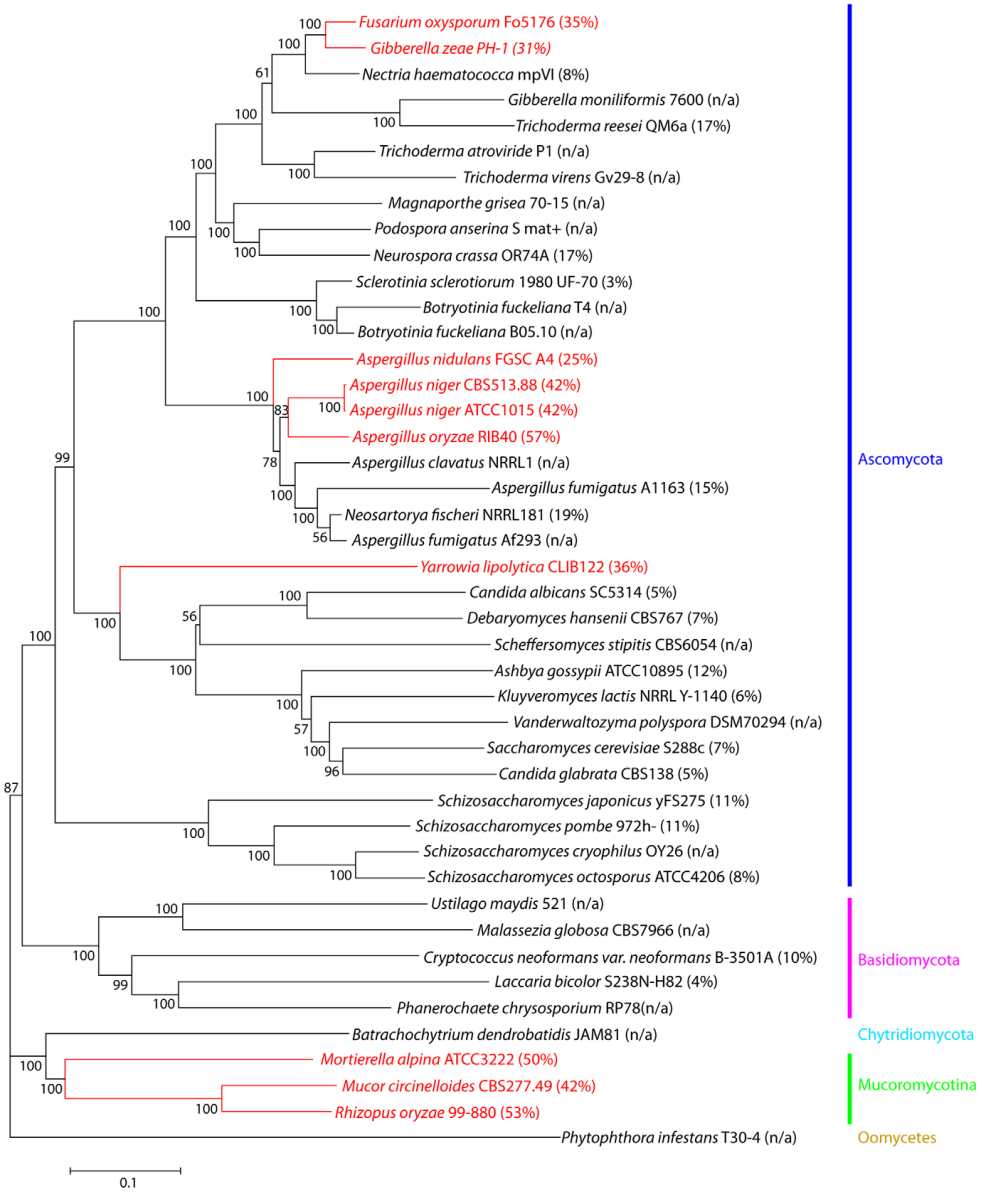
Phylogenetic analysis. Cladogram based on genes involved in fatty acid metabolic pathway. Orthologous proteins were defined as reciprocal best hit proteins with a minimum of 50% identity and 70% of the length of the query protein as calculated by the BLAST algorithm with a threshold value of E≤1×10^−5^. Phylogenies were analyzed based on 12 orthologous proteins identified among 43 genera and inferred from the resulting 98,765 amino character alignment using the Neighbor-Joining method. Numbers on the left are percent of bootstrapping, performed on 1000 replicates with alpha parameters and the fraction of invariant sites estimated once from the original data. *Phytophthora infestans* T30-4 in the Oomycetes was used as the outgroup. Scale bar shows evolutionary distances in replacements per site. The orthologous proteins used for this analysis were carnitine *O*-acetyltransferases, fatty acid desaturase 9, phospholipid∶diacylglycerol acyltransferase, lysophosphatidate acyltransferase, acetyl-CoA carboxylase, 3-oxoacyl-[acyl-carrier-protein] synthase II, fatty acid desaturase 6I and 6II, fatty acid synthase, and 5-aminolevulinate synthase. Numbers in parentheses are reported lipid production in the corresponding fungus. Question marks indicate species in which lipid production is uncertain. Species in red are oleaginous, with lipid production greater than 20%.

To determine a possible evolutional relationship between oleaginous fungi (≥20% lipid/biomass dry weight) and non-oleaginous fungi, twelve orthologous proteins involved in lipid metabolism were identified among 43 genera and used for phylogenetic analysis. The phylogeny segregated fungal species of the Ascomycota, Mucoromycotina and Basidiomycota. *Batrachochytrium dendrobatidis* in the Chytridiomycota was clustered with species in the Mucoromycotina. Furthermore, it placed ten oleaginous fungi into four clusters (**Figure 4**). *M. alpina*, *Rhizopus oryzae* and *Mucor circinelloides*, sharing an average amino acid identity of 79%, were grouped in one cluster. Four *Aspergillus* genera, with an average amino acid identity of 89%, were placed in another. *Fusarium oxysporum* and *Gibberella zeae* (*Fusarium graminearum*) were also congregated with an average amino acid identity of 92%. *Yarrowia lipolytica* was a singleton in a separated branch. These results suggest that oleaginous fungi may have acquired their lipogenic capacity during evolution after the divergence of Ascomycota, Basidiomycota, Chytridiomycota and Mucoromycota.

### Lipogenesis pathway

**Figure 5:**
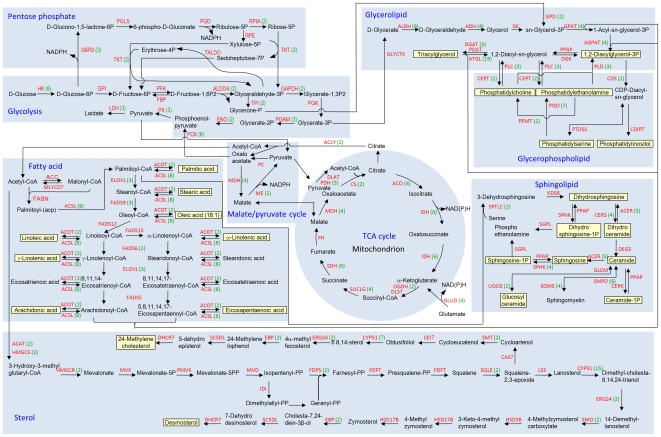
Predicted lipogenesis pathway in *M. alpina.* The glycolysis, pentose phosphate pathway, fatty acid synthesis, tricarboxylic acid cycle, malate/pyruvate cycle, sterol, glycerophospholipid, sphingolipid and glycerolipid synthesis pathways are outlined. Lipids detected by lipidomics are highlighted in yellow. Enzyme names are indicated and followed in parentheses by the number of genes encoding them. Enzymes conventionally thought to occur in a given pathway but for which homologs were not found in *M. alpina* are followed by a question mark. Hexokinase (HK, EC2.7.1.1), glucose-6-phosphate 1-dehydrogenase (G6PD, EC1.1.1.49), 6-phosphogluconolactonase (PGLS, EC3.1.1.31), phosphogluconate dehydrogenase (PGD, EC1.1.1.44), ribose 5-phosphate isomerase (RPIA, EC5.3.1.6), ribulose-5-phosphate-3-epimerase (RPE, EC5.1.3.1), transketolase (TKT, EC2.2.1.1), transaldolase (TALDO, EC2.2.1.2), glucose-6-phosphate isomerase (GPI, EC5.3.1.9), phosphofructokinase (PFK, EC2.7.1.11), fructose-bisphosphatase (FBP, EC3.1.3.11), aldolase A fructose-bisphosphate (ALDOA, EC4.1.2.13), glyceraldehyde 3-phosphate dehydrogenase (GAPDH, EC1.2.1.12), phosphoglycerate kinase (PGK, EC2.7.2.3), triose-phosphate isomerase (TPI, EC5.3.1.1), phosphoglycerate mutase (PGAM, EC5.4.2.1), enolase (ENO, EC4.2.1.11), pyruvate kinase (PK, EC2.7.1.40), lactate dehydrogenase (LDH, EC1.1.1.27), phosphoenolpyruvate carboxykinase (PCK, EC4.1.1.32 and EC4.1.1.49), acetyl-CoA carboxylase (ACC, EC6.4.1.2), malonyl-CoA decarboxylase (MLYCD, EC4.1.1.9), fatty acid synthase (FASN, EC2.3.1.86), acyl-CoA synthetase (ACSL, EC6.2.1.3), acyl-CoA thioesterase (ACOT, EC3.1.2.27), fatty acid elongase (ELOVL, EC2.3.1.-), fatty acid delta 9 desaturase (FADS9, EC1.14.19.1), fatty acid delta 12 desaturase (FADS12, EC1.14.19.6), fatty acid delta 15 desaturase (FADS15, EC1.14.19.-), fatty acid delta 6 desaturase (FADS6, EC1.14.19.3), fatty acid delta 5 desaturase (FADS5, EC1.14.19.-), malate dehydrogenase (MDH, EC1.1.1.37), malic enzyme (ME, EC1.1.1.40), pyruvate carboxylase (PC, EC6.4.1.1), ATP-citrate lyase (ACLY, EC2.3.3.8), pyruvate dehydrogenase (PDH, EC1.2.4.1), dihydrolipoamide acetyltransferase (DLAT, EC2.3.1.12), citrate synthase (CS, EC2.3.3.1), aconitase (ACO, EC4.2.1.3), isocitrate dehydrogenase (IDH, EC1.1.1.41 and EC1.1.1.42), glutamate dehydrogenase (GLUD, EC1.4.1.2 and EC1.4.1.3), 2-oxoglutarate dehydrogenase (OGDH, EC1.2.4.2), dihydrolipoamide succinyltransferase (DLST, EC2.3.1.61), succinyl-CoA ligase (SUCLG, EC6.2.1.4 and EC6.2.1.5), succinate dehydrogenase (SDH, EC1.3.5.1), fumarase (FH, EC4.2.1.2), acetyl-CoA acetyltransferase (ACAT, EC2.3.1.9), hydroxymethylglutaryl-CoA synthase (HMGCS, EC2.3.3.10), 3-hydroxy-3-methylglutaryl-CoA reductase (HMGCR, EC1.1.1.34), mevalonate kinase (MVK, EC2.7.1.36), phosphomevalonate kinase (PMVK, EC2.7.4.2), mevalonate pyrophosphate decarboxylase (MVD, EC4.1.1.33), isopentenyl diphosphate isomerase (IDI, EC5.3.3.2), farnesyl diphosphate synthase (FDPS, EC2.5.1.10), farnesyl-diphosphate farnesyltransferase (FDFT, EC2.5.1.21), squalene epoxidase (SQLE, EC1.14.99.7), cycloartenol synthase (CAS, EC5.4.99.8), delta24-sterol methyltransferase (SMT, EC2.1.1.41), cycloeucalenol cycloisomerase (CEI, EC5.5.1.9), sterol 14-demethylase/cytochrome p450 family 51 (CYP51, EC1.14.13.70), delta14-sterol reductase/(ERG24, EC1.3.1.70), cholestenol isomerase/emopamil binding protein (EBP, EC5.3.3.5), sterol C5 desaturase (SC5DL, EC1.14.21.6), 7-dehydrocholesterol reductase (DHCR7, EC1.3.1.21), lanosterol synthase (LSS, EC5.4.99.7), C4 sterol methyl oxidase (SMO, EC1.14.13.72), hydroxysteroid (3-beta) dehydrogenase (HSD3B, EC1.1.1.170), hydroxysteroid (17-beta) dehydrogenase (HSD17B, EC1.1.1.270), serine palmitoyltransferase (SPTLC, EC2.3.1.50), 3-ketodihydrosphingosine reductase (KDSR, EC1.1.1.102), alkaline ceramidase (ACER, EC3.5.1.23), ceramide synthetase (CERS, EC2.3.1.24), sphingolipid delta-4 desaturase/degenerative spermatocyte homolog (DEGS, EC1.14.-.-), phosphatic acid phosphatase (PPAP, EC3.1.3.4), ceramide kinase (CERK, EC2.7.1.138), sphingomyelin phosphodiesterase (SMPD, EC3.1.4.12), sphingomyelin synthase (SGMS, EC2.7.8.27), UDP-glucose ceramide glucosyltransferase (UGCG, EC2.4.1.80), glucosylceramidase (GLCM, EC3.2.1.45), sphingosine kinase (SPHK, EC2.7.1.91), sphingosine-1-phosphate lyase (SGPL, EC4.1.2.27), CDP-diacylglycerol synthase (CDS, EC2.7.7.41), CDP-diacylglycerol inositol 3-phosphatidyltransferase (CDIPT, EC2.7.8.11), phosphatidylserine synthase (PTDSS, EC2.7.8.8), phosphatidylethanolamine methyltransferase (PEMT, EC2.1.1.17), phosphatidylserine decarboxylase (PISD, EC4.1.1.65), choline/ethanolamine phosphotransferase (CEPT, EC2.7.8.1), phospholipase C (PLC, EC3.1.4.3), glycerate kinase (GLYCTK, EC2.7.1.31), aldehyde dehydrogenase (ALDH, EC1.2.1.3), alcohol dehydrogenase (ADH, EC1.1.1.2), glycerol kinase (GK, EC2.7.1.30), glycerol-3-phosphate dehydrogenase (GPD, EC1.1.1.8), glycero-3-phosphate acyltransferase (GPAT, EC2.3.1.15), 1-acylglycerol-3-phosphate acyltransferase (AGPAT, EC2.3.1.51), phospholipase D (PLD, EC3.1.4.4), diacylglycerol kinase (DGK, EC2.7.1.107), diacylglycerol acyltransferase (DGAT, EC2.3.1.20), phospholipid diacylglycerol acyltransferase (PDAT, EC2.3.1.158), triglyceride lipase (GL, EC3.1.1.3).

Because of its high capacity to produce lipid, we focused on *M. alpina*'s lipid metabolic pathway. Based upon the genome information, a lipogenesis pathway was constructed, which mapped the utilization of glucose, generation of acetyl-CoA and NADPH, and biosynthesis of fatty acids, glycerolipids, glycerophospholipids, sphingolipids and sterols (**Figure 5**). *M. alpina* can grow on glucose as the single carbon source. The glycolysis pathway provides pyruvate, a key precursor for acetyl-CoA. The pentose phosphate pathway generates NADPH, a critical reductant for fatty acid synthesis, and other precursors needed for amino acid and nucleic acid biosynthesis. Most enzymes in the glycolysis and pentose phosphate pathways are encoded by 1 to 3 genes, except for hexokinase (HK) and phosphoenolpyruvate carboxykinase (PCK) which are each encoded by 6 genes (**Figure 5**). HK phosphorylates glucose to produce glucose-6-phosphate, the first step in most glucose metabolism pathways. PCK catalyzes the formation of phosphoenolpyruvate from oxaloacetate, and phosphoenolpyruvate is then converted by pyruvate kinase to pyruvate. Two genes encode a type of PCK that is GTP-dependent (EC4.1.1.32) and four genes encode another type of PCK that is ATP-dependent (EC4.1.1.49).

Among the enzymes directly involved in fatty acid synthesis, acetyl-CoA carboxylase (ACC) converts acetyl-CoA to malonyl-CoA, and this conversion is a rate-limiting step in fatty acid biosynthesis. Fatty acid synthase (FASN) carries out enzymatic reactions necessary for the synthesis of SAFA [typically palmitic acid (PA, 16:0)] from acetyl-CoA and malonyl-CoA. Fatty acid delta 9 desaturase (FADS9) introduces the first double bond into SAFA producing MUFA. Fatty acid elongases (ELOVL) extend fatty acid carbon chains and other fatty acid desaturases introduce additional double bonds to generate PUFA. Fatty acids can exist as a free form or an acyl-CoA form, interconverted by acyl-CoA thioesterase (ACOT) and acyl-CoA synthetase (ACSL). There is one gene each encoding ACC, FASN, fatty acid delta 5 desaturase (FADS5), fatty acid delta 12 desaturase (FADS12) and omega-3 desaturase (FADS15). There are two genes encoding fatty acid delta 6 desaturase (FADS6), three for FADS9, three for ELOVL and two for ACOT; however, ACSL is encoded by six genes (**Figure 5**). A homolog for malonyl-CoA decarboxylase (MLYCD), which counters ACC action, was not identified. If *M. alpina* has no enzyme with MLYCD function, this could increase malonyl-CoA levels, thus favoring lipid synthesis.

**Figure 6:**
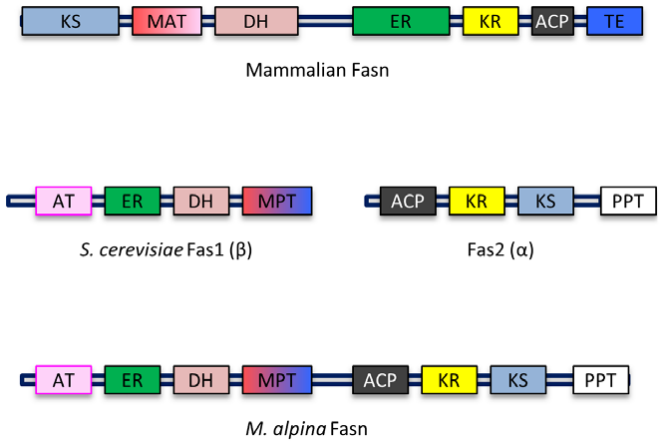
FASN domain comparison. Enzymatic domain organization of mammalian, *S. cerevisiae* and *M. alpina* Fasn is shown. Note: *S. cerevisiae* Fasn comprises two genes, Fas1 and Fas2, encoding two peptides. AT: acetyltransferase, ER: β-enoyl reductase, DH: dehydratase, MAT: malonyl-CoA-/acetyl-CoA-ACP transacylase, MPT: malonyl/palmitoyl transferase, ACP: acyl carrier protein, KR: β-ketoacyl reductase, KS: β-ketoacyl synthase, PPT: phosphopantetheine transferase, TE: thioesterase. Domains are not drawn to scale.

Interestingly, in *M. alpina*, FASN is encoded as a single polypeptide with an acetyltransferase, β-enoyl reductase, dehydratase, malonyl/palmitoyl transferase, β-ketoacyl reductase, β-ketoacyl synthase, phosphopantetheine transferase activity and an acyl carrier protein domain (**Figure 6**).

The tricarboxylic acid (TCA) cycle is important in generating ATP, NAD(P)H, and citrate. TCA derived citrate can be converted to acetyl-CoA through the action of ATP-citrate lyase (ACLY). The enzymes in the TCA cycle are encoded by two or more genes, with the exception of fumarase (FH) (**Figure 5**). Three genes encode a type of isocitrate dehydrogenase (IDH, EC1.1.1.41) that generates NADH, and three genes encode another type of IDH (EC1.1.1.42) that generates NADPH. Similarly, two genes encode a type of glutamate dehydrogenase (GLUD, EC1.4.1.2) that produces NADH and two genes encode another type of GLUD (EC1.4.1.3) that generates NADPH.

Besides the TCA cycle, there are two other major sources of NADPH: the pentose phosphate pathway, as mentioned earlier, and the malate/pyruvate cycle (**Figure 5**). Two malic enzyme genes were identified in *M. alpina*: one is identical to the gene coding for isoforms III/IV, which is presumed to be cytosolic and provides NADPH [20], [21], and the other is homologous to the malic enzyme gene coding for isoform II in *Mucor circinelloides*, which is not considered to be associated with fatty acid biosynthesis, but rather, is involved in anaerobic metabolism [22].

Enzymes involved in glycerolipid, glycerophospholipid, and sphingolipid synthesis are encoded by various numbers of genes. Most notably, there are 18 genes encoding triglyceride lipase (GL, EC3.1.1.3). Amino acid homology analysis suggests that eleven contain the class 2 lipase domain (pfam01674), one contains the class 3 lipase domain (pfam01764), three contain the PGAP1-like domain (pfam07819), two contain the CVT17 domain (COG5153), and one contains the DUF2424 domain (pfam10340).

Sterols can be synthesized from acetyl-CoA. Enzymes involved in this pathway are encoded by either 1 or 2 genes each (**Figure 5**). Fifteen cytochrome P450 genes were identified in *M. alpina*. Amino acid homology analysis suggests that their products are distantly related to the enzymes in family 51, which in other organisms have sterol 14-demethylase activity. Interestingly, homologs for cycloartenol synthase (CAS, EC5.4.99.8) and cycloeucalenol cycloisomerase (CEI, EC5.5.1.9) were not found.

### Major lipid products

To confirm the lipid products predicted from our gene pathway analysis (**Figure 5**), total fatty acids, glycerolipids, glycerophospholipids, sphingolipids and sterol lipids were determined. *M. alpina* was cultured at 25°C and 12°C for 6 days. As expected, it synthesized a large quantity of lipid, representing approximately 45% of dry mycelial weight, and more than 50% of the fatty acids were AA (**Table 3**). There were no significant temperature-associated differences in the levels of SAFA or omega-6 PUFAs. Interestingly, omega-3 PUFA accumulation was 40-fold higher in cultures grown at 12°C than at 25°C (**Table 3**).

**Table 3:** Total fatty acid distribution*.

Glucose (g/L)	Yest extract (g/L)	KNO3 (g/L)	Temperature (°C)	SAFA						MUFA								ω6 PUFA					ω3 PUFA					
				14:0	16:0	18:0	20:0	22:0	24:0	14:1	16:1	total 18:1	oleate	vaccenate	20:1	22:1	24:1	18:2	20:2	18:3 n-6	20:3	20:4	18:3 n-3	20:5	22:5	22:6	24:5	24:6
20	5	10	25	0.4±0.1	**13.4**±1.8	**11.3**±2.9	0.8±0.1	1.5±0.5	0.8±0.1		0.1±0	**8.1**±0.6	7.7±0.7	0.4±0	0.3±0.1		0.1±0.1	**7.9**±1.0	0.5±0.1			**54.8**±6.9		0.1±0				
		0		0.3±0.2	**8.5**±1.9	**6.6**±0.6	0.7±0	1.8±0.6	1.5±0.6			**5.2**±1.5	4.8±1.4	0.4±0.1	0.2±0		0.3±0.2	**5.6**±1.3	0.5±0.1			**68.8**±6.1						
20	5	10	12	1.4±1.0	**14.8**±5.7	**10.1**±3.9	0.8±0.2	1.6±0.5	1.2±0.4		0.2±0.1	**13.4**±5.1	12.6±4.8	0.8±0.4	0.7±0.1		1.0±0.6	**4.3**±1.4	0.5±0.1	0.1±0		**47.7**±18	0.1±0.1	**2.4**±0.5				
		0		0.8±0.4	**11.5**±3.8	**9.2**±1.1	0.7±0	1.2±0.1	0.7±0.1		0.1±0.1	**13.7**±5.9	13.2±5.7	0.5±0.2	0.7±0.1		1.1±0.2	**3.2**±0.5	0.5±0.1			**54.3**±11	0.1±0.1	**2.1**±0.5				

*Molar percent of each fatty acid in day 6 mycelia. Averages ± standard deviations are shown. Blank: undetectable. 14:0 myristic acid (tetradecanoic acid), 14:1 myristoleic acid (tetradecenoic acid), 16:0 palmitic acid (hexadecanoic acid), 16:1 palmitoleic acid (hexadecenoic acid), 18:0 stearic acid (octadecanoic acid), 18:1 oleic acid (9Z-octadecenoic acid) and vaccenic acid (11Z-octadecenoic acid), 18:2 linoleic acid (octadecadienoic acid), 18:3 α-linolenic acid (9Z,12Z,15Z-octadecatrienoic acid) and γ-linolenic acid (6Z,9Z,12Z-octadecatrienoic acid), 20:0 arachidic acid (eicosanoic acid), 20:1 gondoic acid (eicosenoic acid), 20:2 (Eicosadienoic acid), 20:3 dihomo-γ-linolenic acid (eicosatrienoic acid), 20:4 arachidonic acid (eicosatetraenoic acid), 20:5 EPA (eicosapentaenoic acid), 22:0 behenic acid (docosanoic acid), 22:1 erucic acid (docosenoic acid), 22:5 DPA (docosapentaenoic acid), 22:6 DHA (docosahexaenoic acid), 24:0 lignoceric acid (tetracosanoic acid), 24:1 nervonic acid (tetracosenoic acid), 24:5 (tetracosapentaenoic acid), 24:6 (tetracosahexaenoic acid).

**Figure 7:**
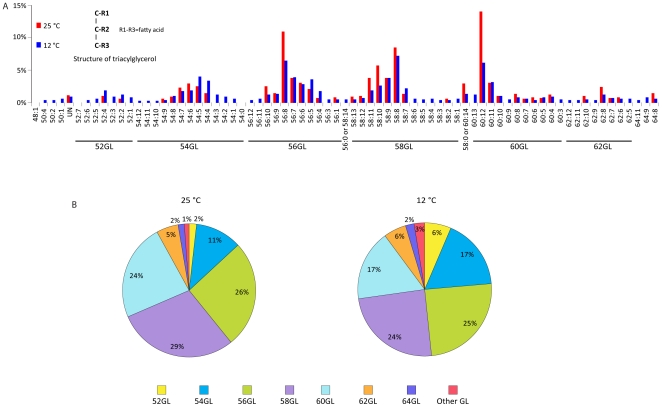
Glycerolipid distribution. Major glycerolipid (GL) species detected in *M. alpina* cultures grown at 12°C and 25°C (**A**) and percent distribution (**B**) are shown. The number before the colon indicates the total number of carbons in the three fatty acyl chains, and the number after the colon represents the total number of double bonds (e.g. 56:8 represents several species with a combined number of fatty acyl carbon atoms of 56 and 8 double-bonds).

One outstanding characteristic of *M. alpina* is the efficient synthesis of glycerolipids, which are non-polar species stored as lipid droplets primarily in the form of triacylglycerols. According to our analysis, *M. alpina* synthesized more than 400 different triacylglycerols with various fatty acyl groups arranged on the glycerol moiety. Major species of triacylglycerols are shown in **Figure 7**. AA was the most common fatty acids incorporated into triacylglycerols, with a ratio of approximately 0.6–1.1 mol per mol triacylglycerols (ratios over 1 reflect the incorporation of more than one AA chain in a single triacylglycerol molecule). The most prominent triacylglycerol species were 56:8GL [which likely contained one palmitic acid (16:0) and two arachidonic acid (20:4) chains on the glycerol backbone] and 60:12GL [which likely contained three arachidonic acid chains].

**Figure 8:**
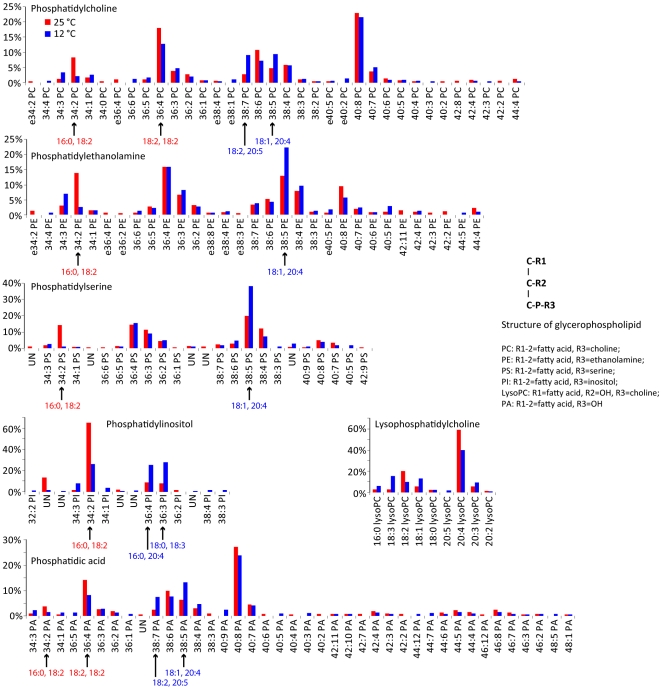
Glycerophospholipid distribution. Phosphatidylcholine (PC), phosphatidylethanolamine (PE), phosphatidylserine (PS), phosphatidylinositol (PI), phosphatidic acid (PA), and lysophosphatidylcholine (LysoPC) species distributions are shown. Arrows indicate the major differences in glycerophospholipid between cultures at 12°C and 25°C.

There are four major types of glycerophospholipids: phosphatidylcholine (PC), phosphatidylethanolamine (PE), phosphatidyl-serine (PS) and phosphatidylinositol (PI). PC, PE, PS, PI and intermediate products, phosphatidic acid (PA) and lysophosphatidylcholine (lysoPC) were detected, but not diphosphatidylglycerol (**Figure 8**). Multiple species of PC, PE, PS, PI and PA were identified with various fatty acyl groups arranged on the *sn-1* and *sn-2* position of the glycerol backbone (**Figure 8**). Overall, between 0.4 and 0.6 mole of AA was incorporated per mole of glycerophospholipids. The molar ratios of AA in each type of glycerolipid were as follows: 0.6–0.7 in PC, 04–0.6 in PE, 0.3–0.7 in PS, 0.2 in PI, 0.6–0.8 in PA and 0.5 in LysoPC. Interestingly, an increased incorporation of highly unsaturated PUFAs into glycerophospholipids was observed at 12°C compared to 25°C (**Figure 8**). It is likely that this change, which results in an increased desaturation of glycerophospholipids, is an adaptation to enhance the plasma membrane fluidity at lower temperatures.

**Figure 9:**
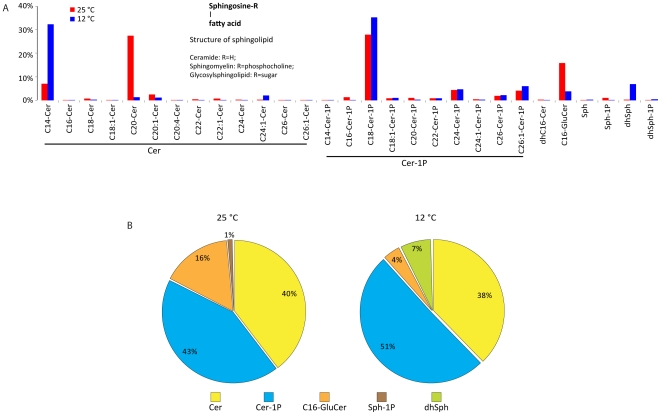
Sphingolipid distribution. Sphingolipid species (**A**) and percent distribution (**B**) are shown. Cer: ceramide; Cer-1P: ceramide-1-phosphate; GluCer: glucosyl ceramide; Sph: sphingosine; Sph-1P: sphingosine-1-phosphate; dhSph: dihydrosphingosine; dhSph-1P: dihydrosphingosine-1-phosphate. Numbers in parentheses are percent of total sphingolipids.

*M. alpina* produced various species of ceramides and ceramide-1Ps, which comprised more than 80% of the sphingolipids (**Figure 9**). Other sphingolipids were also detectable, including sphingosine, sphingosine-1P, dihydrosphingosine and dihydrosphingosine-1P, but not sphingomyelin (**Figure 9**).

In contrast to the complexity of glycerolipids, glycerophospholipids and sphingolipids, only two sterol lipids, desmosterol and 24(28)-methylene-cholesterol, were detected. Collectively, lipid profile results support the predicted lipogenesis pathway.

## Discussion

Microbial oil is a great source of energy and dietary fat. In the present study, we have assembled the first draft genome of *M. alpina*, partially mapped its lipogenesis pathway, and determined its major fatty acid, glycerolipid, glycerophospholipid, sphingolipid and sterol species.

*M. alpina* is one of many oleaginous fungi. It is unclear how oleaginous fungi evolved. Our phylogenic result, based upon lipid metabolic genes, is consistent with the phylogeny constructed on the basis of 440 core orthologs [23]. It suggests that the evolutionary history of lipid metabolic genes was largely dominated by vertical inheritance rather than lateral gene transfer. Our analysis also placed ten oleaginous fungi into four clusters, indicating that oleaginous fungi may have acquired their lipogenic capacity during evolution. In addition, the divergence level of lipid metabolic genes is lower than that of core orthologs. For instance, within the cluster of *Schizosaccharomyces* genus, the average amino acid identity is 64% for core orthologs [23] and 79% for lipid metabolic genes (our study). The data suggests that lipid metabolic genes have a lower mutation rate than the average rate of core orthologs. A low divergence of genes is indicative of their essentiality for the survival of organism.

One noticeable feature of the *M. alpina* genome is its high percentage (50%) of genes in multigene families, a feature shared by all three highly oleaginous fungi in the Mucoromycotina subphylum (**Table 2**). In the predicted *M. alpina* lipogenesis pathway (**Figure 5**), approximately 60% of genes are in multigene families. The number of genes encoding each enzyme varies widely.

Cytochrome P450s are heme-thiolate proteins involved in the oxidative degradation of various compounds. They can be divided into 4 classes, according to the method by which electrons from NAD(P)H are delivered to the catalytic site. Primary amino acid sequence identity between different P450s are relatively low, however their secondary structure is conserved. Several thousands of P450s have been identified among fungi [24]. Our study identified 15 cytochrome P450 genes in *M. alpina*. Protein BLAST analysis of *M. alpina* P450s shows homology between P450 family 83A1, 90A, abscisic acid 8′-hydroxylase and other cytochrome P450 related domains. However, it is unclear which cytochrome P450 is involved in sterol synthesis in *M. alpina*. None of the P450 genes in this organism presented strong homology to the cytochrome P450 family 51, which has sterol 14-demethylase activity in other organisms.

Eighteen lipases identified contain either the class 2 lipase, class 3 lipase, PGAP1-like, CVT17 or DUF2424 domain. Lipases with a class 2 or 3 domain may function as glyceride lipases which hydrolyze ester bonds in acylglycerols, releasing diacylglycerol, monoacylglycerol, glycerol and free fatty acids. Lipases with the PGAP1-like domain may work as a GPI inositol-deacylase. This deacylation is important for the efficient transport of GPI-anchored proteins from the endoplasmic reticulum to the Golgi body. Lipases with the CVT17 domain may operate as autophagic enzymes inside the cell vacuoles. The function of lipases with the DUF2424 domain is still unknown.

Cycloartenol synthase (CAS) and cycloeucalenol cycloisomerase (CEI) form cycloartenol and obtusifoliol intermediates in the synthesis of sterol lipids. While homologs to these enzymes were not identified, we cannot exclude their presence in *M. alpina*. It is possible that they have low homologies to proteins reported from other species.

Our lipogenesis pathway shows five enzymes in three different pathways that can generate NADPH (**Figure 5**), a critical reductant for lipid biosynthesis. It is unclear whether some, or all, of these enzymes play significant roles in the generation of NADPH. However, over-expression of malic enzyme (ME) can increase fatty acid synthesis in fungi [20], suggesting that ME may be a major generator of NADPH, and elevation of NADPH level is a mechanism to increase lipid production in fungi.

There are two types of fatty acid synthases: type-I FASN, found in fungi and mammals, are proteins containing several domains with distinct enzymatic activities [25], whereas type-II FASN, found in bacteria and plants, are a group of proteins with a single enzymatic activity, and multiple enzymes are required for fatty acid synthesis [26]. Although *M. alpina* has a type-I FASN gene, which like the mammalian FASN gene [27], [28], encodes a single polypeptide, the organization of the enzyme's domains is very different. Unlike the mammalian counterpart, the organization of domains in *M. alpina* FASN resembles that of the FASN in yeast, such as *S. cerevisiae, S. pombe, and T. languinosus* [29], [30], [31], where the domains are rearranged but on two polypeptides (**Figure 6**). However, the single peptide type-I FASN is not unique to *Mortierella* in the fungal kingdom; *Ustilago maydis*, *Coprinopsis cinerea*, *Laccaria bicolor,* and *Omphalotus olearius* also have a single peptide form of FASN.

*M. alpina* FADS12 and FADS15 catalyze the formation of omega-6 and omega-3 PUFA, respectively. *M. alpina* produces very low amounts of eicosapentaenoic acid (EPA, 20:5n-3) but maintains a high rate of AA synthesis, which in this study, constituted greater than 50% of the total fatty acids. Although the *M. alpina* genome shows extensive gene duplication, this differential capacity cannot be explained by gene numbers since there is only one gene for each desaturase. However, it is possible that expression levels or catalytic activity of FADS12 and FADS15 could account for the difference, as studies have demonstrated that forced expression of FADS15 in *M. alpina* 1S-4 drastically increases EPA production [32], [33]. Omega-6 and omega-3 PUFA are essential fatty acids which humans cannot synthesize *de novo*. Dietary omega-3 fatty acids mainly come from deep water fish. With increasing ocean pollution and decreasing fish populations, there is an urgent need to identify alternative sources of omega-3 PUFA. To this end, *M. alpina* may be engineered to produce omega-3 PUFA efficiently.

Concurrently, there is a worldwide effort in sequencing fungal genomes, however, most of the genomes sequenced (33/43) are in the Ascomycota phylum (**Table 2**). Our study will be the second published genome in the Mucoromycotina which, together with *Rhizopus oryzae* genome [34], will be useful for better characterization of fungi in this subphylum.

In summary, genome sequencing, pathway mapping and major lipid profiling have, for the first time, provided a comprehensive picture of lipid metabolism in *M. alpina*. It will be important to determine how gene expression is regulated during the lipogenesis in *M. alpina*, and how it is coordinated between the generation of precursor molecules such as pyruvate and acetyl-CoA, the production of reductant NADPH, and the synthesis of lipids. We are currently conducting transcriptome analysis is to address these issues.

## Materials and Methods

### Fungal culture

*Mortierella alpina* (#32222, American Type Culture Collection, Manassas, Virginia) was inoculated on PDA plates (BD DifcoTM Potato Dextrose Agar cat# 213400) and incubated for 20–30 days at 25°C. Five ml broth (20 g/L Glucose, 5 g/L Bacto yeast extract BD Biosciences cat# 212750, 1 g/L KH_2_PO_4_, 0.25 g/L MgSO_4_, 10 g/L KNO_3_) were added to three plates. Spores were gently scraped off the surface with a sterile loop, and then filtrated through a 40 micron cell strainer. Spores were concentrated by centrifuging at 12,000× g for 15 min, suspended in a small volume of broth, enumerated using a hemocytometer, and kept at −80°C in 30% glycerol at a density of approximately 10^7^ spores/ml. Alternatively, three ml of unconcentrated spore suspension were directly added into 45 ml broth without KNO_3_ in a 250-ml flask covered with 8 layers of cheese cloth, and shaken at 200 rpm, 25°C for 5 days. Cultures were blended using a Braun hand blender for 5 sec/pulse, 8 pulses, then 0.3 g wet mycelia were inoculated into 45 ml broth without KNO_3_ in a 250-ml flask and shaken at 200 rpm, 25°C for 24 h. The above step was repeated once, by which time the whole fungal culture was in proliferative phase and ready for experiments. Mycelia were collected by filtration and weighed. Samples were snap-frozen in liquid nitrogen, pulverized and kept at −80°C until analysis.

### Genome sequencing and analysis

#### Isolation of genomic DNA

Genomic DNA of *M. alpina* was extracted using a modified protocol from Murray and Thompson [35]. Mycelia of *M. alpina* were ground to a fine powder with liquid nitrogen prior to extraction. The powder was gently dispersed in extraction buffer [1.4 M NaCl, 2% cetyltrimethyl ammonium bromide (CTAB), 100 mM Tris-HCl (pH 8.0), 20 mM EDTA, and 1% 2-mercaptoethanol] with a ratio of 10 ml buffer per gram of mycelia. The mixture was incubated for 30 min at 60°C with occasional gentle mixing. The extract was emulsified by gentle inversion with an equal volume of chloroform/isoamyl alcohol (24∶1). After centrifugation at 12,000× g for 10 min, the aqueous phase was removed with a large bore pipette. One tenth volume of 10% CTAB, 1 M NaCl was added and incubated for 10 min at 60°C. The chloroform/isoamyl alcohol treatment was repeated. RNase was added to a final concentration of 100 µg/ml and incubated in a 65°C water bath for 30 min. The DNA sample was extracted with an equal volume of phenol/chloroform/isoamyl alcohol (25∶24∶1), and then with chloroform/isoamyl alcohol (24∶1). DNA was precipitated with one tenth volume of 3 M sodium acetate (pH 5.5) and 2.5 volumes of absolute ethanol and was kept at −80°C for one h. After spinning at 12,000× g at 4°C for 30 min, the pellet was washed with 70% v/v ethanol, briefly dried and dissolved in an appropriate volume of TE buffer.

#### Sequencing and assembly

High molecular weight DNA was used to construct plasmid libraries with inserts of 3–8 kb and fosmid libraries with inserts of 35–40 kb. In total, 92 Mb high quality paired-end data (Phred Q20) was generated by AB 3730 Sanger sequencing platform from the plasmid and fosmid libraries. In addition, 490 MB pyrosequencing data with average read length of 207 bp and 710 Mb pyrosequencing data with average read length of 308 bp were obtained using the Roche 454 Genome Sequencer FLX platform. Pyrosequencing reads were assembled by gsAssembler (2.0.00.20) with default parameter settings [36] and assembled contigs were spliced into overlapping fragments of about 5 kb in length accompanied with quality values. Sanger sequencing reads from plasmid and fosmid clones were combined with overlapping fragments from pyrosequencing contigs [37]. The combined data were assembled by Phrap (http://www.phrap.org) using default settings. Resequencing of low quality regions and closing of gaps were performed by walking on plasmid and fosmid clones and by PCR using custom primers designed by Consed [38].

#### Gene prediction and annotation

*Ab initio* gene prediction was performed on the genome assembly by Augustus [39], GlimmerHMM [40], SNAP [41] trained by *M. alpina* ESTs from this study, and by GeneMark [42] without supervised training. A final set of gene models was selected by EVidenceModeler with equal weight for evidence from each gene prediction software [43]. Predicted genes were annotated by BLAST [44] searches against protein databases with E-value 1E-5: NR (www.ncbi.nlm.nih.gov), KOGs and COGs [45], KEGG [46], Swiss-Prot and UniRef100 [47], BRENDA [48], and by InterProScan [49] against protein domain databases with default parameter settings: Pfam [50], PRINTS [51], SMART [52], PROSITE [53], TigrPfam [54], PANTHER [55]. Pathway mapping was conducted by associating EC assignment and KO assignment with KEGG metabolic pathways based on BLAST search results. Multigene family analysis on predicted genes in the *M. alpina* genome and other sequenced fungal genomes was performed using OrthoMCL [56] with E-value 1E-20.

#### Repetitive elements

Repetitive sequences in the genome assembly were identified by searching Repbase [57] using RepeatMasker (http://www.repeatmasker.org) with default parameter settings, and by *de novo* repetitive sequences search using RepeatModeler (http://www.repeatmasker.org/RepeatModeler.html) with default settings.

#### Non-coding RNAs

Transfer RNAs were predicted by tRNAscan [58] using default settings. Ribosomal RNAs were identified by BLAST search with E-value 1E-10 of known rRNA modules in other fungal genomes. Other non-coding RNAs were predicted by searching the Rfam database [59] by Infernal (http://infernal.janelia.org) using default parameter settings.

#### Mitochondrial genome

A preliminary mitochondrial genome sequence was assembled as a single contig from pyrosequencing data using gsAssembler (version 1.1.03.24) with default parameter settings and was compared with mitochondrial genome of *M. verticillata* [19]. Coding sequences, tRNA and rRNA were identified as described above.

#### Other fungal genomes

Genome sequences of *Candida albicans* SC5314 [60], *Rhizopus oryzae* 99–880 [34], *Aspergillus fumigatus* Af293 [61] and A1163 [62], *Aspergillus clavatus* NRRL 1 [62], *Ashbya gossypii* ATCC10895 (*Eremothecium gossypii*) [63], *Aspergillus nidulans* FGSC A4 [64], *Aspergillus niger* CBS 513.88 [65] and ATCC1015 [66], *Aspergillus oryzae* RIB40 [67], *Sclerotinia sclerotiorum* 1980 UF-70, *Botryotinia fuckeliana* B05.10 and T4 [68], *Fusarium oxysporum* Fo5176 [69], *Candida glabrata* CBS138, *Debaryomyces hansenii* CBS767, *Kluyveromyces lactis* NRRL Y-1140 and *Yarrowia lipolytica* CLIB122 [70], *Cryptococcus neoformans var. neoformans B-3501A* [71], *Gibberella zeae* PH-1 (*Fusarium graminearum*) [72], *Laccaria bicolor* S238N-H82 [73], *Malassezia globosa* CBS 7966 [74], *Magnaporthe grisea* 70-15 [75], *Neurospora crassa* OR74A [76], *Podospora anserina* S mat+ [77], *Scheffersomyces stipitis* CBS 6054 (*pichia stipitis* CBS 6054) [78], *Saccharomyces cerevisiae* S288C [79], *Schizosaccharomyces pombe* 972h- [80], *Ustilago maydis* 521 [81], *Vanderwaltozyma polyspora* DSM 70294 [82], *Schizosaccharomyces cryophilus* OY26, *Schizosaccharomyces japonicus* yFS275 and *Schizosaccharomyces octosporus* ATCC4206 [83] were published. Genome sequences of, *Nectria haematococca* MPVI (*Fusarium solani*) (Joint Genome Institute), *Trichoderma atroviride* P1 (Joint Genome Institute), *Trichoderma virens* Gv29-8 (Joint Genome Institute), *Batrachochytrium dendrobatidis* JAM81 (Joint Genome Institute), *Mucor circinelloides* (Joint Genome Institute), *Trichoderma reesei* QM6a (Joint Genome Institute), (Broad Institute), *Phanerochaete chrysosporium* RP78 (Broad Institute), *Neosartorya fischeri* NRRL 181 (Broad Institute), *Gibberella moniliformis* 7600 (*Fusarium verticillioides*) (Broad Institute), *Phytophthora infestans* T30-4 (Broad Institute) were downloaded from the corresponding websites.

### EST sequencing and analysis

An EST library was constructed from *M. alpina* cultured at 25°C and sequencing by the Illumina Genome Analyzer IIx platform, generating 0.64 Gb single-end sequences with read length of 36 bp and 1.14 Gb paired-end sequences with read length of 45 bp. The raw data was filtered by EULER [84] to remove reads and regions of low quality. Transcript sequences assembled by Velvet [85] with K-mer set to 25 were used to evaluate the completeness of the genome assembly by BLAST with E-value 1E-5. PASA [86] was used with default parameter settings to align transcript sequences to the genome sequence and 3,400 transcript sequences with associated gene structures were used to train *ab initio* gene prediction software.

### Lipid analysis

#### Fatty acid analysis

Approximately 20 mg of mycelia were used for each lipid extraction. Accurately weighed portions of pulverized mycelium were extracted using the method of Bligh and Dyer [87] under acidified conditions with pentadecanoic acid and heneicosanoic acid added as internal standards. The solvent from the fungal extract was removed under a stream of nitrogen. Lipids were saponified in 1 ml of freshly prepared 5% ethanolic potassium hydroxide at 60°C for 1 h under an argon atmosphere. After cooling, 1 ml of water was added to the samples and non-saponifiable lipids were extracted into 3 ml of hexane. The aqueous layer was acidified with 220 µl of 6 M hydrochloric acid and the fatty acids extracted into 3 ml of hexane. After removing the hexane in a stream of nitrogen, fatty acids were converted to methyl esters by first treating with 1 ml of 0.5 M methanolic sodium hydroxide at 100°C for 5 min under argon followed by 1 ml of 14% methanolic boron trifluoride at 100°C for 5 min under argon [88]. After cooling the sample was mixed with 2 ml of hexane followed by 4 ml of saturated aqueous sodium chloride. After separating the phases, aliquots of the hexane layers were diluted 24-fold with hexane and then analyzed by GC/MS. One µl was injected in the splitless mode onto a 30 m×250 µm DB-WAXETR column (Agilent Technologies, Santa Clara, California) with 0.25 µm film thickness. The temperature program was as follows: 100°C for 2 min, ramp to 200°C at 16°C per min, hold for one min, ramp to 220°C at 4°C per min, hold one min, ramp to 260°C at 10°C per min, and hold for 11 min. Helium was the carrier gas at a constant flow of 1.5 ml/min. The mass spectrometer was operated in positive-ion electron impact mode with interface temperature 260°C, source temperature 200°C, and filament emission 250 µA. Spectra were acquired from *m/z* 50 to 450 with a scan time of 0.433 s. Lower-boiling fatty acid methyl esters were quantified using the pentadecanoic acid internal standard, whereas higher-boiling methyl esters were quantified using the heneicosanoic acid internal standard.

#### Glycerolipid analysis

Total lipid extracts from 20 mg pulverized mycelium were evaporated under a stream of nitrogen and redissolved in 1 ml of chloroform/methanol (1∶1). Aliquots (50 µl) were evaporated under a stream of nitrogen, solubilized in Triton X-100, and enzymatically assayed for triacylglycerol content as described [89]. For relative quantification of triacylglycerol molecular species, 2 µl or 4 µl aliquots of the lipid extracts were diluted to 1 ml with methanol containing 1 µg/ml sodium formate. Samples were infused at 5 µl/min into the electrospray source of a TSQ triple quadrupole tandem mass spectrometer (Thermo Fisher Scientific, Waltham, Massachusetts). The spray potential was 3.8 kV. Nitrogen was used as the sheath gas and ion sweep gas at 15 and 1.5 psi, respectively. The ion transfer capillary was held at a temperature of 270°C and a potential of 35 V. Spectra of the [M+Na]^+^ ions were averaged over 1 min and corrected for carbon isotope effects. For qualitative identification of triglyceride molecular species the same sample solutions were infused with the same source parameters. The TSQ was programmed to acquire product ion spectra of the 50 most abundant [M+Na]^+^ ions in the sample. The collision gas pressure was 1.5 mTorr; collision energy (CE) was 40 eV. Each product ion spectrum was averaged over 10 s ( = 10 scans). For each sample the product ion spectrum lists were compiled into a single Excel spreadsheet using a modification of the Xcalibur software's export.exe utility (Xcalibur Inc., Vienna, Virginia). Excel functions were then used to interpret the daughter ion spectra. Peaks with *m/z* inconsistent with neutral loss of a free fatty acid were filtered out and then putative fatty acid nominal masses were calculated by rounding and subtraction. Lastly, an Excel macro identified molecular species by searching for sets of three putative fatty acid nominal masses that, together with the mass of the glycerol backbone and sodium, added up to the corresponding precursor ion mass.

#### Glycerophospholipid analysis

Aliquots (50 µl) from the lipid extracts were digested with perchloric acid and assayed for phosphorus as described [90]. Using the lipid phosphorus data, aliquots of each sample amounting to 30 nmol of total phospholipid were analyzed by normal-phase high-performance liquid chromatography-tandem mass spectrometry (LC-MS/MS) using modifications of previously described methods [91], [92]. Samples (70 µl) were injected in chloroform∶methanol (2∶1) onto a μPorasil silica column, 3.9×300 mm; 10 µm particle size (Waters Corporation, Milford, Massachusetts). Phospholipids were eluted at 2 ml/min with the following gradient: 100% hexane/isopropanol/water/ammonium hydroxide (150∶200∶5∶2, v/v) (Solvent A) for 1 min, ramp to 100% hexane/isopropanol/water/ammonium hydroxide (150∶200∶35∶2, v/v) (Solvent B) over 59 min, hold 100% Solvent B for 10 min, and regenerate the column with 100% Solvent A for 20 min. A splitting tee directed 5% of the column effluent to the TSQ mass spectrometer (Thermo Fisher Scientific) for monitoring the elution of phospholipid classes. The remaining 95% of the column effluent was collected for analysis. The following phospholipid classes were detected as [M+H]^+^ ions by scanning in positive-ion mode: PE by common neutral loss of 141 Da with CE = 25 eV; PS by common neutral loss of 185 Da with CE = 22 eV; and choline-containing lipids (PC, lysoPC, and SM) as precursors of *m/z* 184.1 with CE = 35 eV. Phospholipids detected in negative-ion mode as [M-H]^−^ ions were PI, measured as precursors of *m/z* 241 with CE = 48 eV, and PA/PG/cardiolipin, measured as precursors of *m/z* 153 with CE = 40 eV. Scanning was initiated using the Scan Events function of Xcalibur 2.0 software. The spray potential was 4 kV in positive-ion mode and −2.8 kV in negative-ion mode with a nitrogen sheath gas pressure of 10 psi. The collision gas pressure was 0.8 mTorr and the ion transfer capillary temperature 270°C. Each phospholipid subclass was collected, evaporated under a stream of nitrogen, digested with perchloric acid, and then assayed for phosphorus. PA and PC co-eluted in this HPLC system but were separated by subjecting the collected PA/PC fraction to thin-layer chromatography on silica gel G plates developed with chloroform/methanol/ammonium hydroxide (60∶35∶8). PA and PC bands were visualized with iodine vapor, scraped from the plates, digested with perchloric acid, and assayed for phosphorus [90]. A profile mass spectrum was generated for each phospholipid class by averaging the appropriate precursor or neutral loss spectra acquired during the elution. Molecular species were quantified as mol% of the class by correcting the profile spectrum list for carbon isotope and mass-dependent ion throughput effects. For confirmation of molecular species assignments to the peaks in the profile spectrum, fresh aliquots of the lipid extracts were diluted to 10 µM total phospholipid in chloroform∶methanol∶water (49∶49∶2) and infused directly into the electrospray source at 5 µl/min. Samples were analyzed by a profile scan in precursor or neutral loss mode for the appropriate class followed by data-directed acquisition (DDA) product ion scans in negative-ion mode for fatty acyl anion products of the 25 most abundant ions in the profile scan. Product ion data were exported to Excel and interpreted in a manner analogous to that described above for triglycerides.

#### Sterol lipid analysis

Non-saponifiable lipid fractions prepared as described above were evaporated under a stream of nitrogen then redissolved in 175 µl hexane for GC-MS analysis. Samples were analyzed on a 30 m×250 µm (0.25 µm film thickness) HP-5MS column with 1 µl injection volume in the splitless mode. Helium carrier gas was used at constant flow 1.2 ml/min. The temperature program was as follows: 170°C for 1 min, ramp to 280°C at 20°C/min, and then hold for 16 min. Mass spectrometer settings were as described above for fatty acid analysis, except that spectra were acquired from *m/z* 50 to 550 with a scan time of 0.517 s. For the quantification of known sterols, approximately 150 mg fresh weight mycelia were saponified in the presence of 25 µg of 5α-cholestane internal standard. Non-saponifiable lipids were extracted into 1 ml of hexane and analyzed on GC with flame ionization detection using a 6890N GC-FID (Agilent Technologies). Samples (1 µl) were injected directly onto a ZB-50 column 15 m×530 µm, 1 µm film thickness (Phenomenex, Torrance, California). The column was maintained at 250°C throughout the 15-min run. Hydrogen carrier gas was set at constant head pressure of 6 psi (flow rate approx. 15 ml/min). Nitrogen was the makeup gas, and the detector temperature was 280°C.

#### Sphingolipid analysis

Analyses of ceramide species and sphingosine were performed on a Thermo Finnigan TSQ 7000, triple-stage quadrupole mass spectrometer (Thermo Fisher Scientific) operating in a Multiple Reaction Monitoring positive ionization mode as described [93], [94]. Samples were fortified with the internal standards and extracted into a one-phase solvent system with ethyl acetate/isopropanol/water (60∶30∶10%, v/v). 4 ml was separated followed by evaporation under nitrogen. After reconstitution in 100 µl of acidified (0.2% formic acid) methanol, samples were injected on the HP1100/TSQ 7000 LC/MS system and gradient-eluted from the BDS Hypersil C8, 150×3.2 mm, 3-µm particle size column, with 1.0 mM methanolic ammonium formate/2 mM aqueous ammonium formate mobile phase system. Peaks corresponding to the target analytes and IS were collected and processed using the Xcalibur software. Final results were calculated as the level of the particular SPLs normalized by sample weight and expressed as SPLs/g.

## Supporting Information

Table S1 **Summary of genomic data.**
(DOC)

Table S2 **Summary of repetitive sequences.**
(DOC)
