# Supplementary material for: Genome Characterization of the Oleaginous Fungus Mortierella alpina
Source: PLoS One. 2011 Dec 8;6(12):e28319. doi: 10.1371/journal.pone.0028319 (PMC3234268; doi:10.1371/journal.pone.0028319)
Supplement: Table S1 — Summary of genomic data. (DOC) [file pone.0028319.s001.doc]

Supplementary Table 1. Summary of genomic data

| **Type of genomic data** | **Length (Mb)** | **Sequence coverage** |
| --- | --- | --- |
| N50 length of all scaffolds | 1.84 |  |
| N50 length of all contigs | 0.087 |  |
| Length of contigs (number=1028) in scaffolds larger than 2 kb | 37.49 |  |
| Length of singleton contigs (number =826) between 500 bp and 2 kb | 0.89 |  |
| Estimated length of gaps (number =552) in multi-contig scaffolds | 2.32 |  |
| Sanger, 3-8 kb, plasmid | 72.01 | 1.77 X |
| Sanger, 35-40 kb, fosmid | 20.86 | 0.51 X |
| 454 pyrosequencing | 1199.40 | 29.47 X |
| **Total** | **1292.26** | **31.75 X** |
