# Supplementary material for: Genome Characterization of the Oleaginous Fungus Mortierella alpina
Source: PLoS One. 2011 Dec 8;6(12):e28319. doi: 10.1371/journal.pone.0028319 (PMC3234268; doi:10.1371/journal.pone.0028319)
Supplement: Table S2 — Summary of repetitive sequences. (DOC) [file pone.0028319.s002.doc]

Supplementary Table 2. Summary of repetitive sequences

| **Type of repetitive sequences** | **Number of elements** | **Length occupied (bp)** |
| --- | --- | --- |
| SINEs | 173 | 19427 (0.05%*) |
| LINEs | 948 | 114687 (0.30%) |
| LTR elements | 960 | 469099 (1.22%) |
| DNA elements | 183 | 72642 (0.19%) |
| Total classified | 2264 | **675855 (1.76%)** |
| Unclassified | 4458 | 2124998 (5.54%) |
| Total | 6722 | 2800853 (7.30%) |

SINEs: short interspersed elements; LINEs: long interspersed elements; LTR: long terminal repeat; DNA elements: DNA transposons. *percent of genome.
